# Establishment and validation of a clinicopathological prognosis model of gastroenteropancreatic neuroendocrine carcinomas

**DOI:** 10.3389/fonc.2022.999012

**Published:** 2022-09-26

**Authors:** Jing Chen, Yibing Liu, Ke Xu, Fei Ren, Bowen Li, Hong Sun

**Affiliations:** ^1^ Hebei Key Laboratory for Chronic Diseases, School of Basic Medical Sciences, North China University of Science and Technology, Tangshan, China; ^2^ The Third Bethune Clinical Medical College, Jilin University, Changchun, China; ^3^ The Second Bethune Clinical Medical College, Jilin University, Changchun, China

**Keywords:** gastroenteropancreatic neuroendocrine carcinomas, nomogram, overall survival, SEER database, clinicopathologic feature

## Abstract

**Background:**

Gastroenteropancreatic neuroendocrine carcinomas (GEP-NECs) are a rare, highly malignant subset of gastroenteropancreatic neuroendocrine neoplasms (GEP-NENs). However, how to predict the prognosis of GEP-NECs by clinical features is still under study. This study aims to establish and validate a nomogram model of overall survival (OS) in patients with GEP-NECs for predicting their prognosis.

**Methods:**

We selected patients diagnosed with GEP-NECs from the Surveillance, Epidemiology, and End Results (SEER) database and two Chinese hospitals. After randomization, we divided the data in the SEER database into the train cohort and the test cohort at a ratio of 7:3 and used the Chinese cohort as the validation cohort. The Cox univariate and multivariate analyses were performed to incorporate statistically significant variables into the nomogram model. We then established a nomogram and validated it by concordance index (C-index), calibration curve, receiver operating characteristic (ROC) curve, the area under the curve (AUC), and the decision curve analysis (DCA) curve.

**Results:**

We calculated the nomogram C-index as 0.797 with a 95% confidence interval (95% CI) of 0.783–0.815 in the train cohort, 0.816 (95% CI: 0.794–0.833) in the test cohort and 0.801 (95% CI: 0.784–0.827) in the validation cohort. Then, we plotted the calibration curves and ROC curves, and AUCs were obtained to verify the specificity and sensitivity of the model, with 1-, 3- and 5-year AUCs of 0.776, 0.768, and 0.770, respectively, in the train cohort; 0.794, 0.808, and 0.799 in the test cohort; 0.922, 0.925, and 0.947 in the validation cohort. The calibration curve and DCA curves also indicated that this nomogram model had good clinical benefits.

**Conclusions:**

We established the OS nomogram model of GEP-NEC patients, including variables of age, race, sex, tumor site, tumor grade, and TNM stage. This model has good fitting, high sensitivity and specificity, and good clinical benefits.

## Introduction

Neuroendocrine carcinomas (NECs) are a rare, highly malignant subgroup of neuroendocrine neoplasms (NENs), while the gastroenteropancreatic system is one of the most common areas from which NECs can originate (1, 2). With the improvement of diagnostic techniques of the gastroenteropancreatic system, such as the wide use of gastroscopy, colonoscopy, and ultrasonic examination, an increasing number of patients with early gastroenteropancreatic NECs (GEP-NECs) can be diagnosed. Therefore, the incidence of GEP-NECs has increased gradually in recent years (3–5). The incidence of NECs is less well defined due to changes in WHO classification over the past 10 years (6), but epidemiological studies estimate the rate to be approximately 0.4 per 100,000 person-years (5, 7). In the United States, a total of 6,291 cases with GEP-NECs were diagnosed between 1973 and 2012 (8). To get things worse, the prognosis is poor, with a median survival of only 19 months (9, 10). The ever-increasing number of cases and poor prognosis have also pushed researchers to develop better models to better understand the disease.

At present, few studies focus only on GEP-NECs, while more studies concentrate on gastroenteropancreatic neuroendocrine tumors (GEP-NETs), another subtype of gastroenteropancreatic NENs (GEP-NENs) that are less malignant and have more cases and a wider range. It is generally believed that the factors affecting the prognosis of GEP-NETs mainly include the patient’s age, tumor grade, pathological stage, and primary tumor site (1, 11–13). However, the existing models only analyze the influence of a single factor on the prognosis of the disease, so the prediction effect can be very limited. At present, there is no prognostic model for GEP-NECs alone. Since GEP-NECs and GEP-NETs are different subtypes of GEP-NENs, it is questionable whether the prognostic prediction model of GEP-NETs is fully applicable to GEP-NECs. Therefore, we want to take cases from a larger database and integrate these potential prognostic factors to build a better model focusing on GEP-NECs that can be used by a large number of clinicians.

For this purpose, we retrieved and collected the data of patients with GEP-NECs from the Surveillance, Epidemiology, and End Results (SEER) database and two Chinese hospitals and constructed and validated a nomogram based on clinicopathological information.

## Methods

### Database and study population from the SEER database

**Figure 1:**
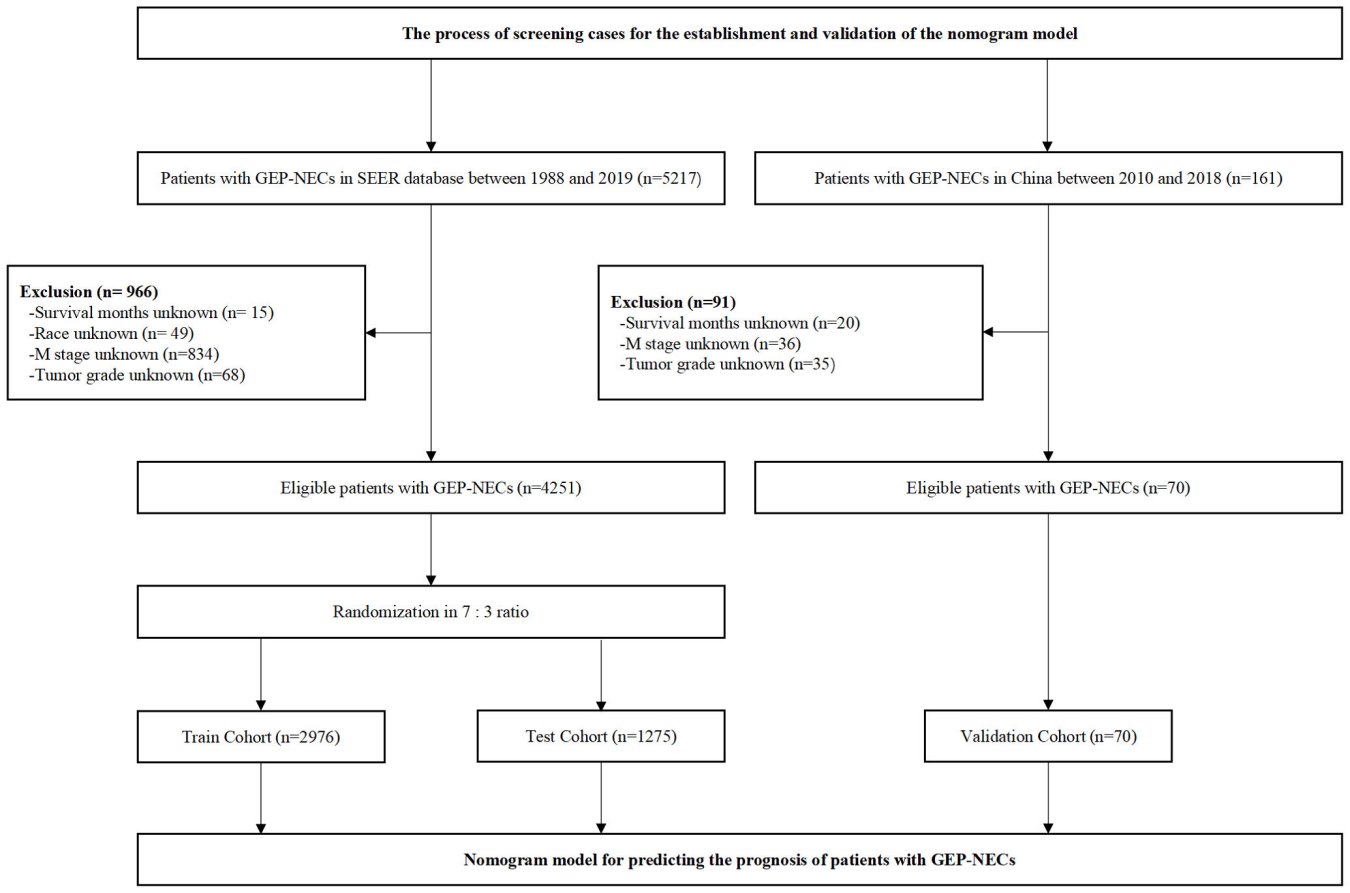
Flow chart of data filtering. SEER, Surveillance, Epidemiology, and End Results; GEP-NECs, gastroenteropancreatic neuroendocrine carcinomas.

We reviewed patients diagnosed with GEP-NECs between 1988 and 2019 from the SEER database, which was established in the United States to provide first-hand information for clinical work worldwide (14, 15). All data were downloaded from SEER*Stat 8.4.0.1. The inclusion and exclusion criteria for the data are as follows 1): They all had a definite diagnosis according to the 2003 WHO diagnostic criteria for NECs 2). Patients with benign tumors or tumors suspicious for malignancy without confirmation were excluded from this study 3). The tumor must have originated in the gastroenteropancreatic system rather than metastatic cancer 4). We excluded patients with unknown survival time, race, M stage, or tumor grade. Through the above criteria, we finally selected 4251 patients to be included in our study and randomly divided the population into a train cohort and a test cohort at a ratio of 7:3. The data filtering process is shown in **Figure 1**.

### Database and study population from two Chinese hospitals

To better verify the applicability of the constructed nomogram model in Chinese patients, we reviewed patients of the Second Hospital of Jilin University and China-Japan Union Hospital of Jilin University between 2010 and 2018. The screening criteria were consistent with those patients from the SEER database, which are described above. Finally, 70 patients with GEP-NECs were selected as the validation cohort. The data filtering process is shown in **Figure 1**.

### Selection of clinical variables

While the prognostic factors of GEP-NECs are still uncertain, prognostic factors of GEP-NETs were used as variables to be included in the nomogram model. Existing studies have shown that older age, male sex, high tumor stage, and low tumor differentiation are markers of poor tumor prognosis, and tumors originating in the pancreas have the worst prognosis (16–23). Therefore, we identified several factors, including age, sex, tumor site, tumor grade, and tumor-node-metastasis (TNM) stage. In the variable of tumor site, we included tumors originating from the large intestine, pancreas, small intestine and stomach, in which the large intestine includes colon, rectum, anus, anal canal and anorectum. In addition, race was also included in the nomogram model as one of the variables, since our data involved different ethnic groups. All the above variables were included in the nomogram model. For clinical outcomes, overall survival (OS) was selected as the endpoint.

### Establishment and validation of the nomogram model

We used IBM SPSS Statistic to perform univariate and multivariate Cox regression models in the train cohort to determine variables that would be output for the establishment of the nomogram model. Once we obtained these variables, we used R software 4.2.1 to build a nomogram model. Then, the test and validation cohorts were used to evaluate the newly established nomogram. The comparison between nomogram prediction and actual observation was assessed by the concordance index (C-index) and the calibration curve. The receiver operating characteristic (ROC) curve and area under the curve (AUC) were used to evaluate the sensitivity and specificity of the model. In addition, we plotted the decision curve analysis (DCA) curve of this nomogram model to verify the clinical benefits. All analyses were completed with R software 4.2.1 and IBM SPSS software, and the analysis was statistically significant only when P<0.05.

## Results

### Clinicopathological data of included cases

According to the inclusion and exclusion criteria, a total of 4251 cases in the SEER database were eventually included in this study, of which 2976 were assigned to the train cohort and 1275 were randomly assigned to the test cohort. Among all patients, 46.72% were younger than 60 years old, 53.28% were older than 60 years old, 53.94% were male, and 46.06% were female; 11.79% were black, 76.55% were white, and 11.67% were other races; 32.96% had large intestine cancer, 20.63% had small intestine cancer, 38.37% had pancreatic cancer, and 8.05% had stomach cancer. No significant differences were found between the train and test cohorts in each contained variable. Furthermore, we also selected 70 cases from the two qualified Chinese hospitals as the validation cohort, among which 52.86% were younger than 60 years old, 47.14% were older than 60 years old, 70% were male, and 30% were female. Since these cases were from the colorectal surgery departments of two hospitals in China, none of the patients were white or black, and the primary tumor site was in the large intestine. All results are shown in **Table 1**.

**Table 1:** Clinicopathological and demographic characteristics of patients with GEP-NECs.

Variable	SEER population						China population	
	Whole population		Train cohort		Test cohort		Validation cohort	
	n	%	n	%	n	%	n	%
**All**	4251	100.00	2976	100.00	1275	100.00	70	100.00
**Age**								
<60	1986	46.72	1388	46.64	598	46.20	37	52.86
≥60	2265	53.28	1588	53.36	677	53.80	33	47.14
**Race**								
Black	501	11.79	353	11.86	148	11.61	0	0
White	3254	76.55	2315	77.79	939	73.65	0	0
Other	496	11.67	308	10.35	188	14.75	70	100.00
**Sex**								
Male	2293	53.94	1619	54.40	674	52.86	49	70.00
Female	1958	46.06	1357	45.60	601	47.14	21	30.00
**Tumor site**								
Large intestine	1401	32.96	1008	33.87	393	30.82	70	100.00
Pancreas	1631	38.37	1142	38.37	489	38.35	0	0
Small intestine	877	20.63	606	20.36	271	21.25	0	0
Stomach	342	8.05	220	7.39	122	9.57	0	0
**Tumor grade**								
Grade I	1869	43.97	1223	41.10	646	50.67	22	31.42
Grade II	525	12.35	361	12.13	164	12.86	6	8.56
Grade III	520	12.23	412	13.84	108	8.47	37	52.86
Grade IV	206	4.85	144	4.84	62	4.86	0	0
Unknown	1131	26.61	836	28.09	295	23.14	5	7.14
**T stage**								
T1	1110	26.11	707	23.76	403	31.61	23	32.86
T2	769	18.09	536	18.01	233	18.27	7	10.00
T3	1061	24.96	781	26.24	280	21.96	13	18.57
T4	510	12.00	372	12.50	138	10.82	16	22.86
Unknown	801	18.84	580	19.49	221	17.33	11	15.71
**N stage**								
N0	2098	49.35	1422	47.78	704	55.22	28	40.00
N1	1360	31.99	959	32.22	401	31.45	11	15.71
N2	137	3.22	100	3.36	9	0.71	18	25.71
N3	13	0.31	12	0.40	1	0.08	13	18.57
Unknown	643	15.13	483	16.23	160	12.55	0	0
**M stage**								
M0	2503	58.88	1682	56.52	821	64.39	38	54.29
M1	1748	41.11	1294	43.48	454	35.61	32	45.71

GEP-NECs, gastroenteropancreatic neuroendocrine carcinomas; SEER, Surveillance, Epidemiology, and End Results.

### Establishment of the nomogram model

**Figure 2:**
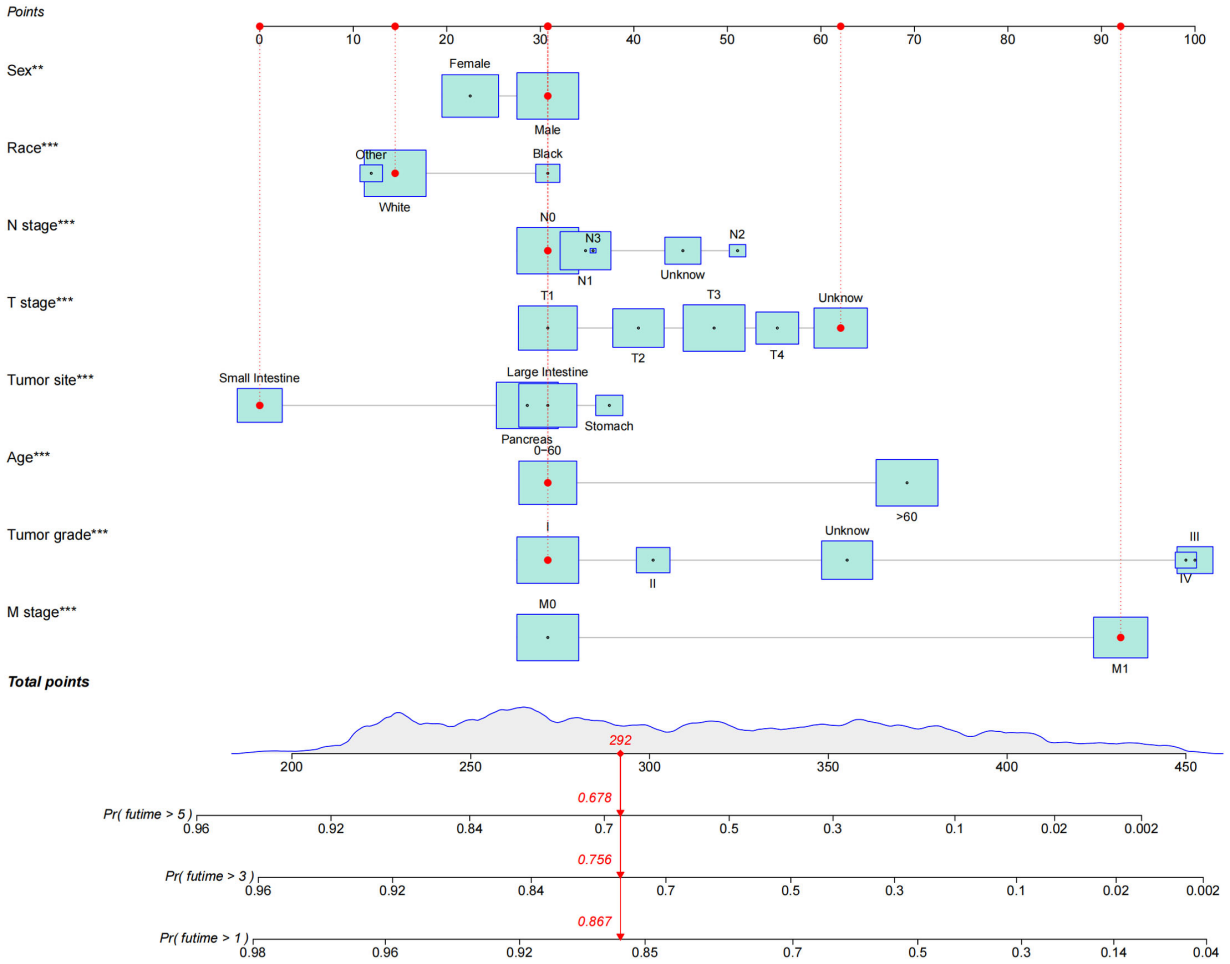
Nomogram of OS established to predict prognosis of patients with GEP-NECs. The red dots and red lines in the figure represent a random example. The patient was a white male younger than 60 years old with small intestine neuroendocrine carcinoma whose T stage was unknown, N stage was N0, M stage was M1, and tumor grade was G1. According to the rotors, the total score of the patients was 292; thus, the 1-, 3- and 5-year survival rates were 86.7%, 75.6%, and 67.8%, respectively.OS, overall survival; GEP-NECs, gastroenteropancreatic neuroendocrine carcinomas. ** P<0.01, *** P<0.001.

The results of univariate and multivariate analyses are shown in **Table 2**. According to the results of univariate Cox analysis, age, sex, tumor site, tumor grade, and TNM stage all showed highly significant differences. In the multifactorial Cox analysis, age, race, sex, tumor site, tumor grade, and TNM stage were all significantly identified. Therefore, all the above variables were incorporated into the models, establishing 1-, 3-, and 5-year nomogram models. The result is shown in **Figure 2**.

**Table 2:** Univariate and multivariate analysis of overall survival in the train cohort.

Character	Univariable analysis			Multivariable analysis		
	HR	95% CI	P-value	HR	95% CI	P-value
**Age**						
< 60	Reference			Reference		
≥60	2.379	2.144-2.640	< 0.001	2.076	1.866-2.309	< 0.001
**Race**						
Black	Reference			Reference		
White	1.131	0.967-1.322	0.122	0.733	0.625-0.859	< 0.001
Other	0.911	0.729-1.139	0.416	0.698	0.557-0.874	< 0.001
**Sex**						
Male	Reference			Reference		
Female	0.821	0.744-0.906	< 0.001	0.854	0.772-0.944	< 0.001
**Tumor site**						
Large intestine	Reference			Reference		
Pancreas	1.047	0.936-1.172	0.415	0.959	0.835-1.100	0.553
Small intestine	0.580	0.500-0.672	< 0.001	0.556	0.469-0.660	< 0.001
Stomach	1.124	0.922-1.370	0.244	1.133	0.925-1.387	0.226
**Tumor grade**						
Grade I	Reference			Reference		
Grade II	1.641	1.362-1.976	< 0.001	1.238	1.025-1.497	0.026
Grade III	6.420	5.539-7.440	< 0.001	3.728	3.161-4.397	< 0.001
Grade IV	6.725	5.482-8.250	< 0.001	3.660	2.943-4.552	< 0.001
Unknown	3.467	3.041-9.954	< 0.001	1.838	1.593-2.120	< 0.001
**T stage**						
T1	Reference			Reference		
T2	1.989	1.649-2.400	< 0.001	1.202	0.986-1.466	0.068
T3	2.587	2.181-3.066	< 0.001	1.402	1.161-1.693	< 0.001
T4	3.538	2.927-4.277	< 0.001	1.594	1.293-1.966	< 0.001
Unknown	5.360	4.510-6.369	< 0.001	1.814	1.483-2.218	< 0.001
**N stage**						
N0	Reference			Reference		
N1	1.505	1.341-1.688	< 0.001	1.079	0.950-1.226	0.238
N2	2.906	2.300-3.672	< 0.001	1.471	1.138-1.900	0.003
N3	3.289	1.805-5.993	< 0.001	1.096	0.589-2.040	0.771
Unknown	3.075	2.697-3.506	< 0.001	1.316	1.127-1.536	< 0.001
**M stage**						
M0	Reference			Reference		
M1	4.152	3.744-4.604	< 0.001	3.206	2.840-3.618	< 0.001

Univariate and multivariate Cox regression models were used to calculate HR and 95% CI for patients with GEP-NECs in the train cohort.

HR, hazard ratio; 95% CI, 95% confidence interval; GEP-NECs, gastroenteropancreatic neuroendocrine carcinomas.

### Nomogram validation

**Figure 3:**
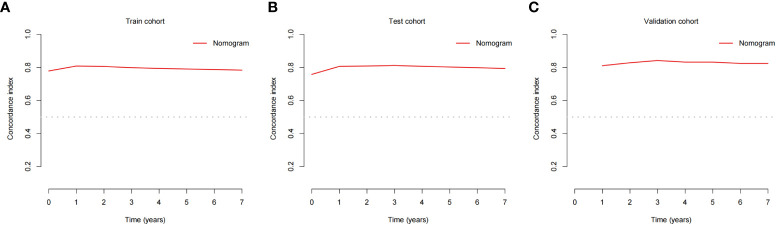
C-indexes for the nomogram in patients with GEP-NECs. **(A)** C-index for the nomogram in the train cohort. **(B)** C-index for the nomogram in the test cohort. **(C)** C-index for the nomogram in the validation cohort. C-index, concordance index; GEP-NECs, gastroenteropancreatic neuroendocrine carcinomas.

**Figure 4:**
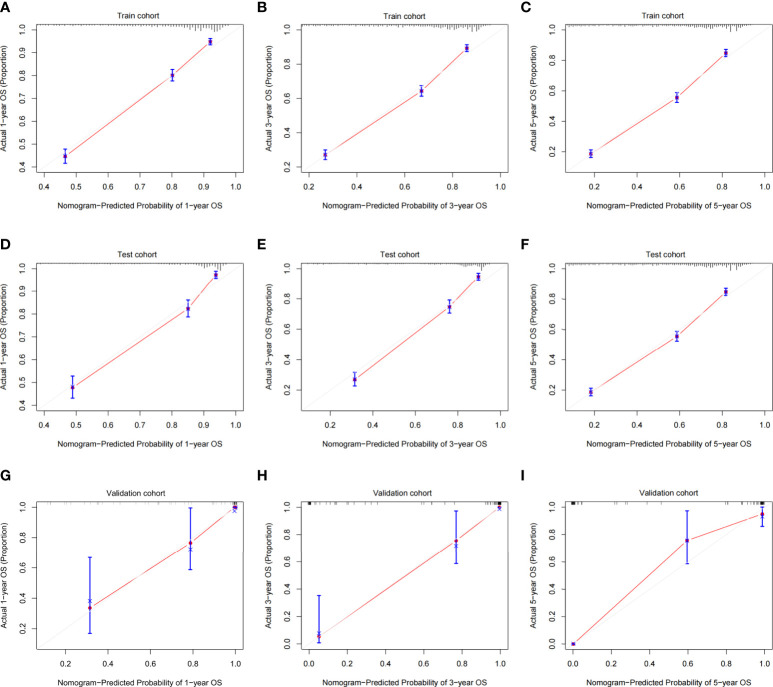
Calibration plots of the OS nomogram model. **(A)** 1-year calibration plot of OS using the train cohort. **(B)** 3-year calibration plot of OS using the train cohort. **(C)** 5-year calibration plot of OS using the train cohort. **(D)** 1-year calibration plot of OS using the test cohort. **(E)** 3-year calibration plot of OS using the test cohort. **(F)** 5-year calibration plot of OS using the test cohort. **(G)** 1-year calibration plot of OS using the validation cohort. **(H)** 3-year calibration plot of OS using the validation cohort. **(I)** 5-year calibration plot of OS using the validation cohort. OS, overall survival.

**Figure 5:**
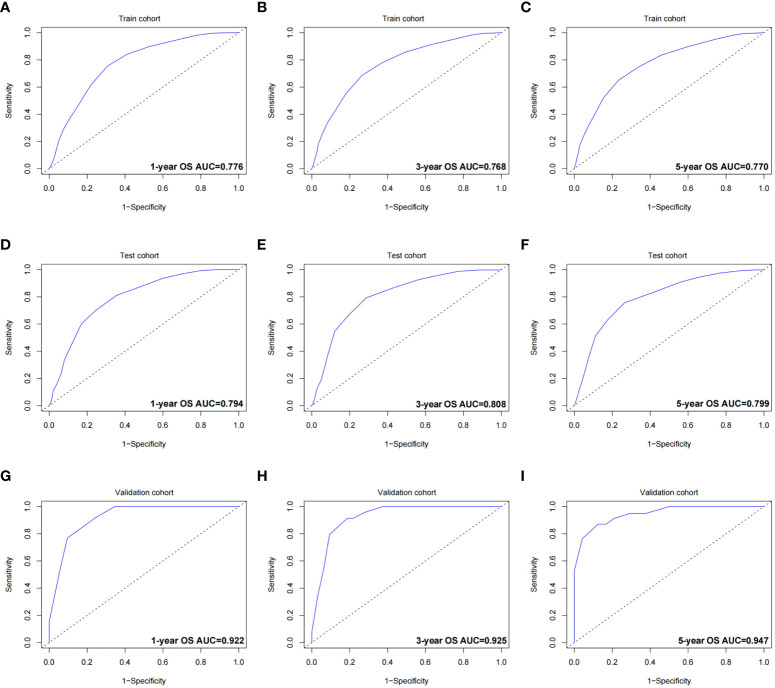
ROC curves of the OS nomogram. **(A)** 1-year ROC curve of the OS nomogram using train cohort. **(B)** 3-year ROC curve of the OS nomogram using train cohort. **(C)** 5-year ROC curve of the OS nomogram using train cohort. **(D)** 1-year ROC curve of the OS nomogram using the test cohort. **(E)** 3-year ROC curve of the OS nomogram using the test cohort. **(F)** 5-year ROC curve of the OS nomogram using the test cohort. **(G)** 1-year ROC curve of the OS nomogram using the validation cohort. **(H)** 3-year ROC curve of the OS nomogram using the validation cohort. **(I)** 5-year ROC curve of the OS nomogram using the validation cohort. ROC, receiver operating characteristic curve; OS, overall survival; AUC, area under the curve.

**Figure 6:**
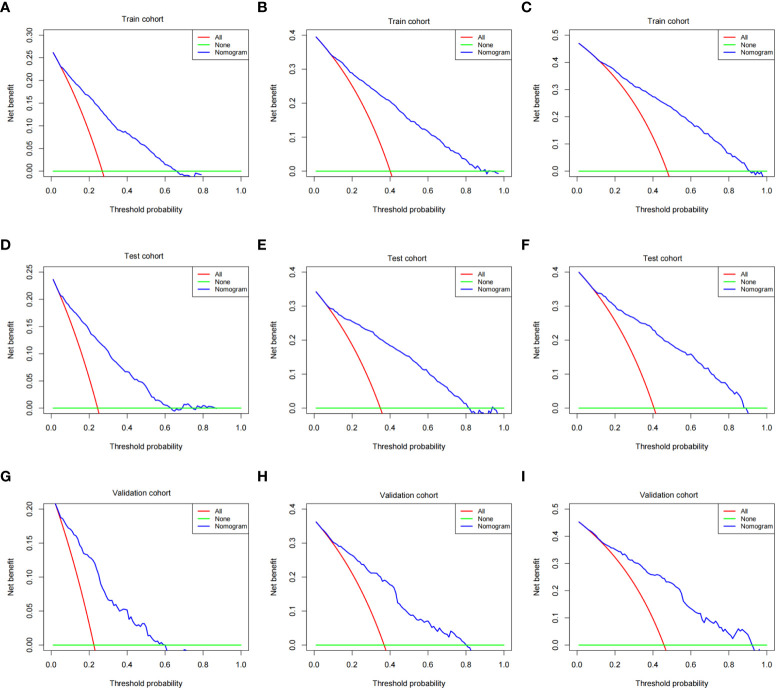
DCA of the OS nomogram. **(A)** 1-year DCA of the OS nomogram using train cohort. **(B)** 3-year DCA of the OS nomogram using train cohort. **(C)** 5-year DCA of the OS nomogram using train cohort. **(D)** 1-year DCA of the OS nomogram using the test cohort. **(E)** 3-year DCA of the OS nomogram using the test cohort. **(F)** 5-year DCA of the OS nomogram using the test cohort. **(G)** 1-year DCA of the OS nomogram using the validation cohort. **(H)** 3-year DCA of the OS nomogram using the validation cohort. **(I)** 5-year DCA of the OS nomogram using the validation cohort. DCA, decision curve analysis; OS, overall survival.

The validation process was carried out internally and externally using the C-index and calibration curves as validation tools. Specifically, the C-index of the OS nomogram was 0.797, with a 95% confidence interval (95% CI) of 0.783–0.815 in the train cohort and 0.816 (95% CI: 0.794–0.833) in the test cohort, as shown in **Table 3** and **Figure 3**. At the same time, the calibration curve showed that the prediction results of the OS nomogram model were of high quality (**Figure 4**). Next, to verify the sensitivity and specificity of the nomogram model, we performed ROC analysis of the OS nomogram, as shown in **Figure 5**. From the figure, we can see that the 1-, 3- and 5-year AUCs in the train cohort are 0.776, 0.768, and 0.770, respectively, while they are 0.794, 0.808, and 0.799 in the test cohort, which shows that the model has high sensitivity and specificity. To further verify the clinical benefit of the nomogram, DCA was carried out on OS. The result is shown in **Figure 6**. In the DCA curves, the nomogram for OS showed good clinical benefit.

**Table 3:** C-indexes for the nomogram in patients with GEP-NECs.

Survival		SEER population				China population	
		Train cohort		Test cohort		Validation cohort	
		HR	95% CI	HR	95% CI	HR	95% CI
**OS**	**Nomogram**	0.797	0.783-0.815	0.816	0.794-0.833	0.801	0.784-0.827

R software was used to calculate the C-indexes of train, test, and validation cohort in the nomogram model.

C-index, concordance index; GEP-NECs, gastroenteropancreatic neuroendocrine carcinomas; OS, overall survival; HR, hazard ratio; 95% CI, 95% confidence interval; SEER, Surveillance, Epidemiology, and End Results.

The same process was also performed in the validation cohort. The C-index was 0.801 (95% CI: 0.784-0.827) in the nomogram model (**Table 3** and **Figure 3**). The calibration curve also shows that the prediction results of the OS nomogram model are of high quality (**Figure 4**). For the ROC curve and AUC, we obtained 1-, 3-, and 5-year AUCs of 0.922, 0.925, and 0.947, respectively (**Figure 5**). Furthermore, the DCA curves also show that the nomogram for OS has good clinical benefits. (**Figure 6**).

## Discussion

According to the above description, we constructed a nomogram model based on age, race, sex, tumor site, tumor grade, and TNM stage, and the validation showed that the model fitting effect was good. Regardless of the train cohort, the test cohort, or the validation cohort, the C-index was greater than 0.7, and the slope of the calibration curve was close to 1. This indicates that the constructed nomogram model has a good prediction effect and is close to the actual situation. For the ROC curve, the AUCs were all greater than 0.7, showing higher accuracy and specificity of the model. Meanwhile, the DCA curves showed good clinical benefit in all three cohorts. The above verification process proves that the nomogram model constructed has a good fitting effect, and all the introduced variables can be used as prognostic factors for GEP-NECs, which is that male sex, older age, high tumor stage and grade are markers of poor tumor prognosis. The results are also similar to existing research (24), which also established a nomogram model for predicting the prognosis of rectal NECs and found that age, sex, TNM stage and grade of the tumor can be clinical prognostic factors.

At present, due to the rarity of GEP-NECs, few studies have focused on the prognosis of GEP-NECs alone and the construction of a prognosis prediction model, while papers have concentrated more on the construction of prediction models using larger datasets of GEP-NETs. The study by Zi-Han Xu et al. included GEP-NETs in the SEER database and constructed a nomogram model based on age, sex, race, marital status, tumor grade and stage, radiotherapy, chemotherapy, and surgery, obtaining the C-indexes for OS prediction in the nomogram as 0.893 (95% CI, 0.883–0.903) and 0.880 (95% CI, 0.866–0.894), respectively, in the train cohort and validation cohort, with the AUCs of the nomogram predicting the 3-year and 5-year OS rates as 0.908 and 0.893, respectively, which shows an effective function of prediction (25). The variables included and the predicted effects in this study are similar to our research results, which can also prove the validity of our research results and the feasibility of clinical application. Similar studies have been performed on patients with GEP-NETs with similar predictive effects (26, 27). These studies are based on a much larger cohort of patients with GEP-NETs, while our study focused on a subset of GEP-NENs with higher malignancy and shorter survival — GEP-NECs — which has more clinical guiding significance in the specific part of patients.

Currently, with the continuous expansion of genomic information, research using genomic information to construct a prognostic model is emerging. The study by Nobuyoshi Takizawa et al. proved the similarity between colorectal neuroendocrine carcinomas and adenocarcinoma by gene sequencing analysis (28). Shinichi yachida et al. confirmed the genetic similarity between large cell and small cell neuroendocrine carcinoma of the pancreas through immunohistochemical and exogenous targeted sequencing (29). Moritz jesinghaus et al. proved that colorectal mixed adenocarcinoma neuroendocrine carcinoma and neuroendocrine carcinoma are genetically closely related to colorectal adenocarcinoma through gene sequencing (30). Gene technology is also widely used not only in the research of neuroendocrine tumors but also in other disease fields (31–33). The advantage of prediction models related to genomic information is that they can more accurately combine certain diseases with a single gene to achieve the purpose of precise treatment. If a less expensive and time-consuming clinical prediction model (such as the nomogram model) could be used to narrow down the range of related genes, this may kill two birds with one stone.

Based on the above, it may be possible to integrate clinicopathological information and genomic information into model construction in the future. Through this model, we can correlate clinicopathological information and genetic information that affect the prognosis of diseases to achieve accurate control of diseases and treat diseases from the root.

To our knowledge, this study is one of the largest and most recent studies focusing on GEP-NECs, providing comprehensive epidemiological and survival data for GEP-NECs, constructing a complete nomogram model, and yielding good predictive results. However, there are some limitations to our study. For example, as Ki-67 and the mitotic index, which are absent in the SEER database, are very important for tumor grading (34), these factors are not taken into account in tumor classification. Moreover, our study is a retrospective study, and if it can be combined with a prospective study, the results will be more perfect. Large-scale multicenter studies will be necessary in the future to remedy these problems.

## Conclusion

We established the OS nomogram model of GEP-NEC patients, including variables of age, race, sex, tumor site, tumor grade, and TNM stage. This model has good fitting, high sensitivity and specificity, and good clinical benefits.

## Data availability statement

The original contributions presented in the study are included in the article/supplementary material. Further inquiries can be directed to the corresponding author.

## Author contributions

JC, YL, and HS designed the study. JC and KX performed the research. YL and FR analyzed the data. JC and YL wrote the paper. BL and HS revised the manuscript for final submission. All authors contributed to the article and approved the submitted version.

## Conflict of interest

The authors declare that the research was conducted in the absence of any commercial or financial relationships that could be construed as a potential conflict of interest.

## Publisher’s note

All claims expressed in this article are solely those of the authors and do not necessarily represent those of their affiliated organizations, or those of the publisher, the editors and the reviewers. Any product that may be evaluated in this article, or claim that may be made by its manufacturer, is not guaranteed or endorsed by the publisher.
